# Stochasticity in Protein Levels Drives Colinearity of Gene Order in Metabolic Operons of *Escherichia coli*

**DOI:** 10.1371/journal.pbio.1000115

**Published:** 2009-05-26

**Authors:** Károly Kovács, Laurence D. Hurst, Balázs Papp

**Affiliations:** 1 Institute of Biochemistry, Biological Research Center, Szeged, Hungary; 2 Department of Biology and Biochemistry, University of Bath, Bath, United Kingdom; University of Pittsburgh, United States of America

## Abstract

In bacterial genomes, gene order is not random. This is most evident when looking at operons, these often encoding enzymes involved in the same metabolic pathway or proteins from the same complex. Is gene order within operons nonrandom, however, and if so why? We examine this issue using metabolic operons as a case study. Using the metabolic network of *Escherichia coli*, we define the temporal order of reactions. We find a pronounced trend for genes to appear in operons in the same order as they are needed in metabolism (colinearity). This is paradoxical as, at steady state, enzymes abundance should be independent of order within the operon. We consider three extensions of the steady-state model that could potentially account for colinearity: (1) increased productivity associated with higher expression levels of the most 5′ genes, (2) a faster metabolic processing immediately after up-regulation, and (3) metabolic stalling owing to stochastic protein loss. We establish the validity of these hypotheses by employing deterministic and stochastic models of enzyme kinetics. The stochastic stalling hypothesis correctly and uniquely predicts that colinearity is more pronounced both for lowly expressed operons and for genes that are not physically adjacent. The alternative models fail to find any support. These results support the view that stochasticity is a pervasive problem to a cell and that gene order evolution can be driven by the selective consequences of fluctuations in protein levels.

## Abstract 2

Gene order in some bacterial metabolic operons reflects ordering in the metabolic pathway. That this is true uniquely for operons expressed at low levels highlights the selective importance of fluctuations in protein levels.

## Author Summary

In bacteria, different enzymes from the same metabolic pathway are often encoded within one transcriptional unit, an operon. There is also, we show, a tendency for the enzymes that are needed earlier in the pathway to feature earlier in the operon, so-called colinearity. Why might this be? We test three ideas, one old and two new. The prior suggestion supposes that proteins of genes early in operons will be at a higher dose. Although some operons are like this, in general, we see no relationship of protein dose with colinearity. We also find no evidence that operons that frequently need up-regulation are any more likely to be colinear. A third model is, however, supported. If an operon is rarely expressed, then all the proteins for this part of metabolism can be lost by chance. Rebooting such metabolism is fastest if the operon is colinear. This model predicts, correctly, that colinearity should be more frequent in operons that are expressed at a low level. This result is important for at least two reasons. First, it supports the view that chance events (such as protein loss) within cells are important on a day-to-day basis. Second, it challenges the supposition that natural selection will be weakest on lowly expressed genes. Where chance events are concerned, natural selection can be strong on genes expressed at a low level.

## Introduction

**Figure 1:**
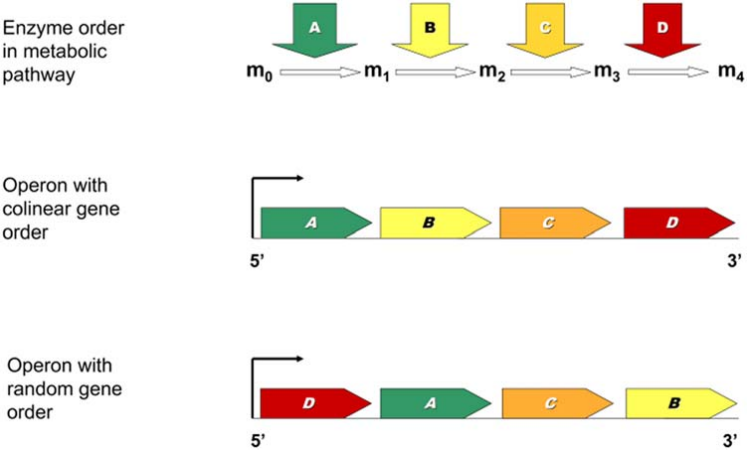
The concept of colinearity between positional sequence of operonic genes and enzymatic steps in a hypothetical metabolic pathway. The first gene arrangement is perfectly colinear, whereas in the second case, two of the six intraoperonic gene pairs have colinear enzymatic orders; therefore, the degree of colinearity is one-third. m_0_, m_1_, …, m_4_ denote metabolites.

It is well established that the chromosomal distribution of genes is not random, and in many genomes, genes that need to be coexpressed tend to cluster [1]–[4]. The coexpression of adjacent genes can be enforced by the action of bidirectional promoters [5], simultaneous opening and closing of chromatin [6],[7], transcriptional spill-over [8], or by inclusion within the same operon [9],[10]. Given that prokaryotic operons generally contain functionally related genes that need to be expressed together [3], gene order evolution in bacteria is often considered to be driven by coexpression (although other scenarios have also been proposed to explain the origin of operons [11],[12]). Such coexpression models do not obviously predict that within an operon there need be selection on gene order. However, a relationship between bacterial morphology and the relative order of genes in a cluster involved in cell division [13] provides some evidence for adaptive gene organization within an operon. Furthermore, a prior comparative genomics study found that horizontal transfer of operonic genes often involves in situ gene displacement by an ortholog from a distant organism without change of the local gene organization [14], hinting at the presence of selection on intraoperonic gene order per se. Nevertheless, it remains unclear whether these phenomena are restricted to certain gene clusters only or whether they could be a more general property of bacterial operons, and most importantly, what selective forces might be responsible for these genomic patterns. Here, then, we ask whether gene order within operons is under selection and if so why? In particular, we investigate whether gene order within metabolic operons of *E. coli* reflect the functional order of the encoded enzymes, i.e., colinearity (Figure 1).

## Results

### Excess of Colinearity in Metabolic Operons

To examine whether gene order within metabolic operons reflect the functional order of the encoded enzymes, we focused on *E. coli*, where high-quality and high-coverage data are available on both biochemical pathways and operon structures. We compiled data on operons encoding at least two enzymes in the same biochemical pathway according to EcoCyc [15], resulting in a list of 70 operons and 321 intraoperonic gene pairs (Methods). For each intraoperonic enzymatic gene pair, we recorded whether their relative position in the operon corresponds to their functional order and hence displays colinearity. Approximately 60% of the 321 gene pairs showed colinearity, compared to 50% expected if intraoperonic gene order was random (*p* = 0.0011, from randomisation, see Methods).

### Theories to Explain Colinearity

At first sight, the above result is unexpected as gene order should not affect the steady-state pathway productivity under the most simplistic scenario (hypothesis 0, see below). To confirm this, we built general mathematical models of operon expression and a linear metabolic pathway with four enzymes (*E_1_…E_4_*) based on previous studies [16],[17]. We used realistic model parameters (see Methods and Table S1) and assigned identical enzyme kinetic parameters and a standard Michaelis-Menten rate law to all four enzymes. Thus, the metabolite concentrations are expressed as follows (Equation 1) for the first three products (*i* = 1...3):
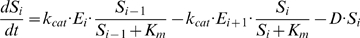
(1)


Where *k_cat_* is the enzyme turnover number, *D* is the dilution rate (i.e., growth rate of the cell), and *K_m_* is the Michaelis constant (see Table S1 for values). Concentration of the first substrate (*S*_0_) was fixed at 1 mM, and the initial concentrations of the other metabolites were set to zero. Metabolic pathway productivity was defined as the amount of end product synthesized during a given time period after operon induction. End product (*S*_4_) formation is given by Equation 2 (dilution of the end product is not considered as we are interested only in the total amount of *S*_4_ synthesized):
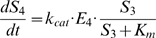
(2)


**Figure 2:**
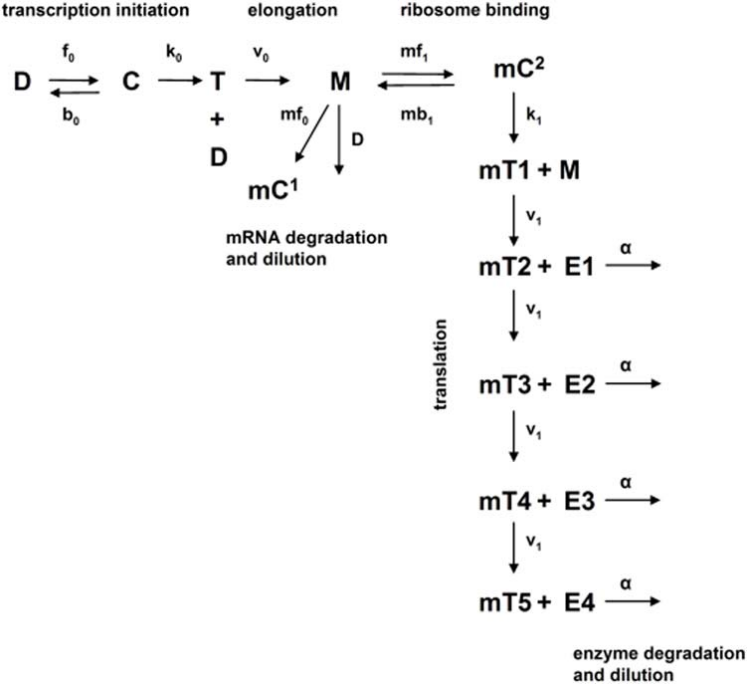
Model of operon expression. Reaction scheme for modelling gene expression of a polycistronic operon. We constructed a model of a four-gene operon and a linear metabolic pathway, containing 5 metabolites and 4 enzymes encoded by the operonic genes. Operon expression was modelled following the “read-through” operon model of Swain [17]. Transcription is modelled as reversible binding of RNAP to promoter (*D*) with rates: *f*_0_ (association) and *b*_0_ (dissociation). Isomerization from closed to open complex and initiation of transcription are approximated as a first-order process (with rate *k*_0_). Only the leader region of the mRNA, *M*, is tracked in the model, which is made by transcribing polymerase, *T*, at rate *v*_0_. mRNA molecules are degraded with rate *mf*_0_, and diluted with rate *D*. Ribosomes compete with degradosomes for leader mRNA and bind reversibly (rates *mf*_1_ for association and *mb*_1_ for dissociation). Translation is started from the *mC* ^2^ state with rate *k*_1_, which then frees *M* for further ribosome or degradasome binding. Enzymes are translated in the *mT* state with rate *v*_1_, and decay and dilute with rate α (α* = D+k_degr_*). In case of a “read-through” operon, only the first cistron has a ribosome binding site; thus, a translating ribosome, *mT*_2_, releases enzyme *E*_1_ before translating the next protein (in the state *mT*_3_). The translation rate parameter was fine tuned to achieve realistic time delays between the appearances of consecutive gene products (approximately 60 s, on average). See Table S1 for parameter values and constants.

Operon expression was modelled following the “read-through” operon model of Swain [17], in which ribosomes move directly from one gene to the next, hence translation events are completely correlated across intraoperonic genes. The formalization includes transcription initiation (RNA polymerase binding and isomerisation), transcription elongation, mRNA degradation and dilution, ribosome binding (ribosomes are bound to the first cistron, hence translation is read-through), translation, and protein degradation and dilution (see Figure 2). The rate of translation was fine tuned to achieve a delay between the appearances of consecutive gene products (*E_i_*) that reflects empirically observed values, i.e., 60 s (see [18],[19]). See Table S1 for model parameters.

Our simulations confirm that at steady state, flux through the pathway is independent of gene order (Table S2). However, the excess of colinear metabolic operons indicates that the above model is missing something. We now consider three hypotheses, one old and two new, that have the potential to explain colinearity. In brief, the hypotheses suppose that colinearity is favoured because (1) it increases productivity associated with higher expression levels of the genes 5′ in operons [20], (2) it provides a transient advantage immediately after up-regulation, and (3) it minimizes metabolic stalling owing to stochastic protein loss.

### Hypothesis 1: Increased Steady-State Flux Due to Higher Expression Levels of 5′ Genes

The first hypothesis [20] proposes that increased productivity of a colinear arrangement could be attributed to a monotonically decreasing mRNA abundance profile along the operon, so-called polarity [21]. That a higher expression level of the first enzyme in the pathway might increase product yield has been experimentally verified in an engineered operon [20]. It has also been shown theoretically that, when the total amount of enzymes within the pathway is fixed (i.e., there is an upper limit of enzyme concentrations), maximal steady-state flux through unbranched pathways can be obtained by a monotonic decrease of enzyme concentrations along the path under some circumstances [22]. In a linear pathway where equilibrium constants of reactions are larger than unity and all enzymes have the same catalytic efficiency, mathematical models predict a decrease of flux control coefficients from the upper end to the lower end of the chain, and therefore, an accumulation of enzyme concentrations at the upper end of the pathway when flux is maximized [22]. If so, and if gene expression levels are not uniformly distributed within operons, then colinearity might confer an advantage by increasing steady-state flux. Indeed, our mathematical model of operon expression coupled with a chain of irreversible enzymatic steps confirms this expectation (Table S3) when the effect of polarity is included in the model (i.e., by introducing degradation of ribosome-bound mRNA intermediates; see Table S3 legend for details). We should then observe higher colinearity in operons with an mRNA abundance profile decreasing from 5′ to 3′.

### Hypothesis 2: Transient Advantage After Up-Regulation

**Figure 3:**
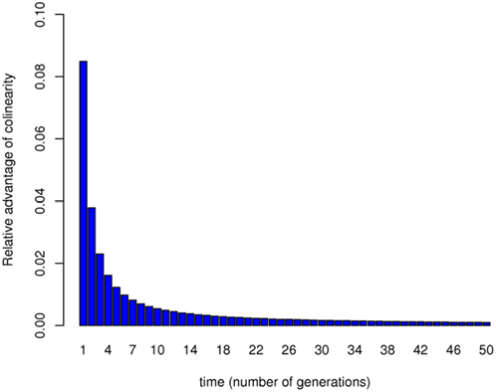
The relative advantage of colinearity decreases with elapsed time after operon induction according to deterministic simulations of the model. Bars represent the relative excess of end product synthesized by a colinear operon (ABCD) compared to an operon with anti-colinear arrangement (DCBA). The operon is induced at *t* = 0. Cell generation time is set to 60 min. See Table S4 for simulation results with different parameter values.

We extend our formalization to identify two further conditions under which colinearity will be under selection (hypotheses 2 and 3). One possibility is that colinearity presents a transient advantage after up-regulation prior to steady state. Consider a simple pathway with two enzymes, A and B. If the order in the operon is AB, then enzyme A can start processing its substrate while B is being synthesized. Conversely, if the order is BA, metabolism cannot start until translation of the second gene is finished, thus slowing the processing of a new metabolite. Our simulations support this possibility (Figure 3 and Table S4). Indeed, a comparison of different simulated intraoperonic gene orders showed that colinearity can increase pathway productivity by up to 8.49% within one cell-generation time following operon induction (comparing the most colinear, ABCD, and least colinear, DCBA, arrangements) owing to the temporal delay between the appearances of consecutive gene products [18],[19], which modulates substrate turnover when total enzyme amount is limited [16],[23]. Moreover, we found that, on average, the effect of swapping the position of two intraoperonic genes depends on their chromosomal distance: swapping adjacent genes had the smallest effect on pathway productivity (see Figure S1).

### Hypothesis 3: Minimized Stochastic Stalling of Metabolism

The above deterministic simulations fail to capture the fact that small numbers of molecules are frequently involved in the process of gene expression and could lead to significant stochasticity in protein abundance [24]. Whereas enzymes encoded in a highly expressed operon are likely to be always present in the cell whenever the operon is induced, stochasticity might play an important role in weakly expressed operons as enzymes could either decay or be diluted by cell division between two expression episodes [25], hence recurrently stalling metabolism. Colinearity could minimize the effect of such stochastic enzyme losses by speeding up the reinitiation of stalled metabolic transformations, in a similar manner as it provides a transient advantage after up-regulation of an inactive pathway (see hypothesis 2).

**Figure 4:**
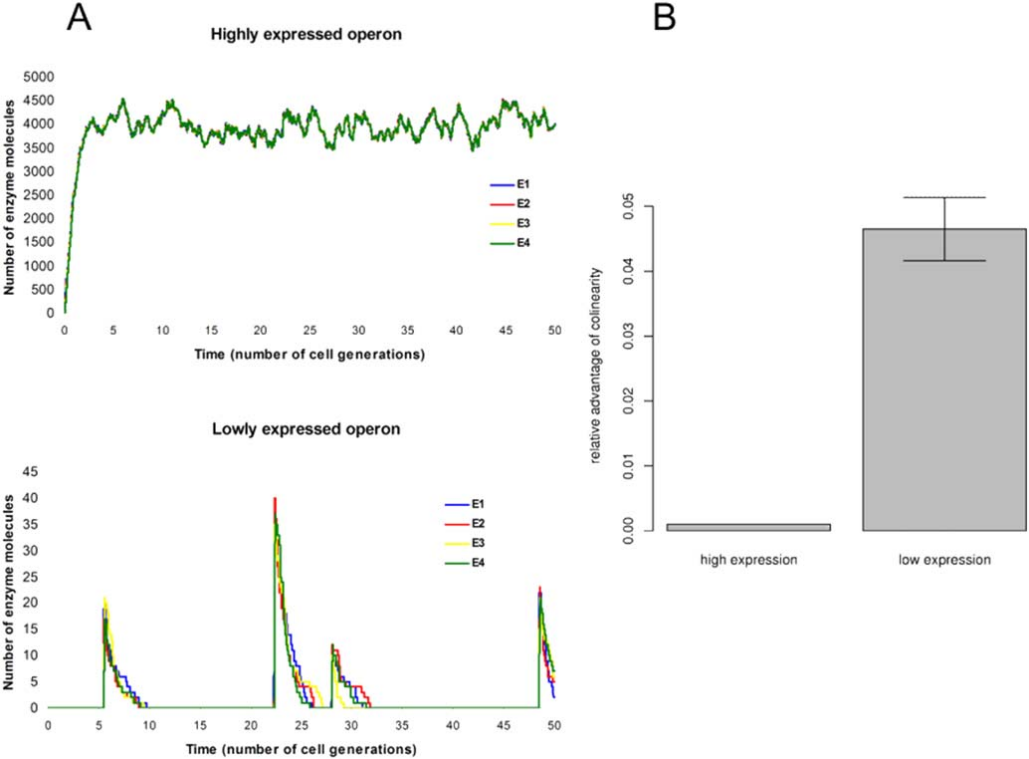
Stochastic simulation results. (A) Temporal fluctuations of enzyme molecule numbers in a lowly and a highly expressed operon (according to stochastic simulations of the model; see Table S1 for model parameters). (B) Calculated average relative advantage of colinearity for a highly and a lowly expressed operon after 50 cell generations (180,000 s) according to stochastic simulations of the model. Mean values of 1,000 repeated simulations and 95% confidence intervals are shown. Colinearity in a lowly expressed operon has a significantly higher advantage than in a highly expressed operon (*p*<2.2×10^−16^, Brunner-Munzel test; a rank-based heteroscedastic method to compare two groups [43]). Error bar indicates 95% confidence interval.

To formally examine this verbal argument, we also simulated stochastically our model to explore how gene order affects pathway productivity as a function of expression level. Different expression levels were simulated by varying the rate of RNA polymerase dissociation from DNA (see Table S1). First, we observe that whereas enzyme molecule numbers fluctuate at both low and high expression levels, enzyme levels frequently drop to zero only when the rate of expression is low (Figure 4A). Importantly, typically all four enzymes are lost between expression bursts at very low transcription rates, which results in the complete stalling of the pathway. Second, we simulated the behaviour of two linear pathways encoded by the same operon, one of which was colinear with the operonic gene order and the other was anti-colinear (thus the metabolic productivity of the two arrangements were directly comparable despite the stochastic nature of the simulations). Pathway performance was assessed after 50 cell-generation times following operon induction. Our analysis showed (Figure 4B) that whereas colinearity in a very lowly expressed operon (mean±standard deviation [SD] protein copy number per cell = 2.4±6.1) can increase pathway productivity by 4.65%, this figure drops to 0.1% for a highly expressed operon (3,959.4±232 protein copies per cell). The effect can be attributed to stochasticity as the advantage of colinearity diminishes when low-expression simulations are run deterministically (i.e., average protein levels are controlled for), and closely resembles the high-expression stochastic simulation scenario with a minute 0.07% increase in pathway performance. Moreover, at low expression levels, even gene orders with an intermediate level of colinearity provide a clear advantage when compared to an anti-colinear arrangement (2.42%, on average).

Thus, based on the above simulations, which appear robust against parameter variations (see Table S5), we expect that colinearity should primarily be a property of lowly expressed operons and of nonadjacent intraoperonic gene pairs. This is an unusual prediction, as more classically, the strength of selection is considered to be greater on more highly expressed genes because these present more opportunity for selection. Such a logic explains, for example, why highly expressed genes evolve slowly [26],[27].

### Empirical Tests of Model Predictions

We can test the three theoretically viable hypotheses by reference to the data on which operons show colinearity. To test hypothesis 1, we gathered a set of microarray expression data on wild-type *E. coli* grown on glucose minimal medium under aerobic and anaerobic conditions (Methods). First, we asked whether operons in general display a decreasing level of mRNA abundances from the 5′ to 3′ end, as observed in the engineered zeaxanthin biosynthesis operon [20] and in the native lactose operon [21]. Indeed, we found an excess of intraoperonic gene pairs in which the gene located closer to the transcription start site has a higher mRNA abundance compared to those located more downstream (*p*<10^−6^; Methods), although we still see many individual operons in which such a trend cannot be detected. This pattern also holds when only metabolic operons are investigated (*p*<0.03). From the above hypothesis, we would expect colinear arrangement only in those operons in which the 5′ genes have higher transcript levels than those located downstream. We hence asked whether the degree of colinearity differs between operons with and without a significantly decreasing mRNA abundance profiles. Using linear trend analysis [28], we identified 26 and 23 operons showing a significantly decreasing mRNA abundance profile under aerobic and anaerobic conditions, respectively (see Methods). Contradicting the prediction of the hypothesis, however, we failed to find an increased colinearity in these operons, neither using the aerobic (*p* = 0.97) nor the anaerobic (*p* = 0.36) expression dataset.

**Figure 5:**
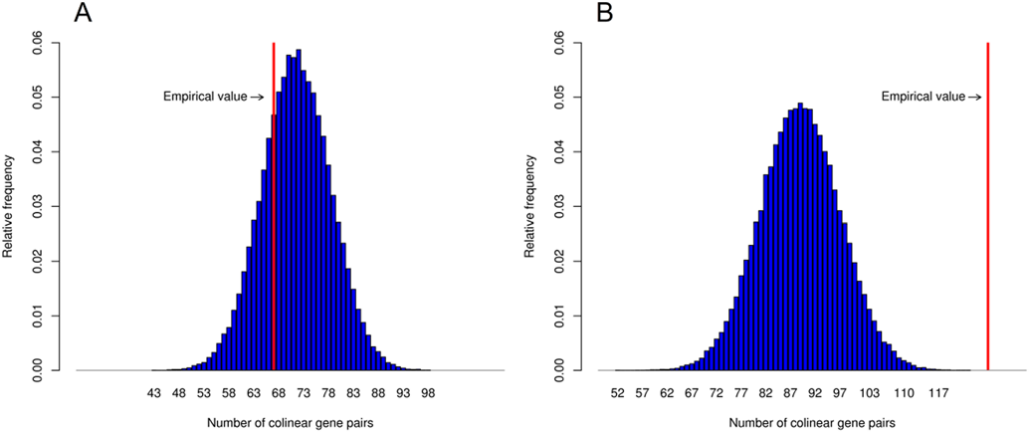
Distribution of the number of colinear gene pairs in randomised samples of highly (A) and lowly (B) expressed operons. Red line indicates the number of colinear gene pairs observed in the *E. coli* genome ([A] 67/143, [B] 127/178 gene pairs). mRNA levels measured under aerobic glucose minimal condition were used to define highly and lowly expressed operons (see Methods). Intraoperonic gene orders were randomised 100,000 times.

To test the prediction of the stochastic stalling hypothesis that colinearity should be found predominantly in lowly expressed operons, we split the set of metabolic operons into two groups based on their mRNA expression levels: operons with average or higher log expression levels and operons with lower than average log expression levels. As predicted, we found (Figure 5) that highly expressed operons have significantly lower degrees of colinearity than those expressed at lower levels (mRNA measured under aerobic condition: *p* = 0.0011, degrees of colinearity: 44.8% vs. 71.6%, anaerobic condition: *p* = 0.0006, degrees of colinearity: 45% vs. 70.8%,; see Methods). Indeed, for the highly expressed operons, there is no significant deviation from null (Figure 5A). It should be noted that in the absence of genome-wide data on protein copy number fluctuations in *E. coli*, we used a population-averaged mRNA level as an inverse proxy for stochasticity in protein concentrations, an assumption that holds in yeast [29] and mammals (L. D. Hurst, unpublished data).

The fact that colinearity is most profoundly seen in low-abundance operons might also be compatible with hypothesis 1 and 2, hence, independent of gene expression noise. First, the advantage of colinearity in the presence of polarity (hypothesis 1) might be more profound in lowly expressed operons. However, simulating the model without stochastic effects predicts the opposite: polarity provides more advantage to colinearity when the expression level is high (Table S3). Furthermore, as explained above, we failed to find any empirical evidence for higher colinearity in operons with significant polarity effects. Second, in hypothesis 2, the variation in expression intensity across conditions is what drives colinearity (i.e., it provides a transient advantage after up-regulation following an environmental shift). Operons that are highly expressed on average might also likely be constitutive in the expression, hence, potentially explaining a connection with abundance. If what matters is the rapid processing of metabolism on up-regulation of the operon, then variation per se may be what matters rather than mean dose. To examine this issue, we used an index of relative variability of mRNA levels measured under 213 conditions as a proxy for gene expression variability across different environments (see Methods). In contrast to expectations, we find that the gene expression level of operons correlates positively, albeit weakly, with their expression variability (*r* = 0.347, *p* = 0.004 and *r* = 0.267, *p* = 0.0268 under aerobic and anaerobic conditions, respectively). Moreover, no significantly increased colinearity can be detected in operons with high expression variability when controlling for expression level, and if there is any trend, it is in the opposite direction (*p* = 0.057 and *p* = 0.128; see Methods). In contrast, the effect of mRNA abundance on colinearity remains significant when expression variability is controlled for (*p* = 0.01 and *p* = 0.016 under aerobic and anaerobic conditions, respectively). We conclude that variation in the environmental specificity of operon expression cannot explain the higher incidence of colinearity in lowly expressed operons.

### Colinearity Depends on Gene Distance

The above mathematical analyses also predict that the impact of gene order rearrangement on metabolic pathway productivity should be most pronounced when the position of genes located distantly within the operon is interchanged (Figure S1). Therefore, one would expect to see more colinearity for distantly located gene pairs compared to those located adjacent in lowly expressed operons. We observe that in support of this expectation, in lowly expressed operons, gene pairs separated by a physical distance of at least one gene length show higher colinearity than those located closer (Fisher exact test, *p*<0.005; the median gene length is 1,070 bp in our dataset). Thus, colinearity is more pronounced for distant enzymatic genes in the operon.

### Intraoperon Metabolite-Level Regulation Does Not Affect Colinearity

All of the above tests presume that if selection favours a given gene order, that order should match the metabolic order. But is it necessarily the case that colinearity is always optimal for metabolic operons? The presence of within-pathway regulatory interactions (i.e., when one enzyme is regulated allosterically or competitively by a product of another enzyme in the same pathway) might impose additional requirements on gene order. If such regulation was more common for abundantly expressed operons, this could explain why colinearity is more common in lowly expressed operons. More specifically, it has been proposed that spatial colocalization of enzymes interacting via small molecule metabolites might enable faster feedback regulation and could be achieved by a closer physical proximity of the enzyme-coding genes within the operon [30]. Thus, there might be selection to place genes of interacting enzymes close to each other in the operon even if they are not colinear with the metabolic pathway.

To test this possibility, we collected data on metabolite-level within-pathway enzymatic interactions from EcoCyc [15] and from a published dataset [31] based on the BRENDA database [32] (Methods). In contrast to the above prediction, we found that the observed average gene distance between interacting enzyme pairs was not significantly different from that expected by chance (*p* = 0.234, *n* = 19 gene pairs), suggesting an absence of clustering of metabolically interacting genes in operons. To further investigate whether the presence of intraoperonic regulatory interactions has an effect on the extent of colinearity, we compared the degree of colinearity in operons with known regulatory interactions (11 operons) to the rest of the dataset (59 operons). A randomisation test showed that the degree of colinearity is not lower in the set in which intraoperon regulations have been reported (*p* = 0.089; see Protocol S1). A similar result was obtained when we controlled for expression-level differences between the two groups (*p* = 0.35, for both aerobic and anaerobic conditions; see Protocol S1). Thus, we failed to find evidence in support of the idea that within-pathway intraoperon metabolic regulation has an influence on gene order and might interfere with colinearity.

## Discussion

To our knowledge, the present study on *E. coli* metabolic operons provides the first systematic evidence that intraoperonic gene order is not random, but rather correlates with the functional order of the encoded enzymes. This is true, however, exclusively for lowly expressed operons, an otherwise curious result given that we usually expect selection to be strongest on highly expressed genes. Our analyses did not find support to the ideas (1) that colinear gene arrangement might be an adaptation to enable high steady-state pathway flux as a result of a decreasing mRNA abundance along the operon [20], or (2) that colinearity presents a transient advantage following up-regulation of the operon in a changed environment. In contrast, the evidence supports the hypothesis that colinearity minimizes stochastic stalling of metabolism at low expression levels: constitutively, but lowly expressed operons are under stronger selection for optimal order as gene expression occurs in random episodes [25], and enzymes from prior expression events might have been lost by decay or by cell division, potentially stalling metabolism. Colinear organization of operonic gene order could minimize any such stalling. This result underscores both the importance of stochastic events to cellular functioning and provides a further case history in which gene order appears to be an adaptation to ensure resilience to stochasticity [33].

An issue that we have not addressed is the potential role of horizontal transfer in establishing colinearity. According to the selfish operon hypothesis [11], novel metabolic functions can be gained by horizontal transfer that moves sets of genes in unison. This hypothesis, the validity of which is questionable as an explanation for which genes reside in operons [4],[10], does not, as far as we can tell, have any specific prediction as to whether an operon should be colinear or not. Consider a pathway with enzymatics steps A → B → C. If all three genes are needed for successful horizontal transfer, then it should not matter whether the order is ABC, CBA, or any other variant. If transfer of two successive enzyme is selectively favourable (e.g., A and B, but not C), then transfer of AB from the operon ABC should be as viable as transfer of BA from operon BAC. Thus, there is no obvious reason why horizontal transfer of operons should impose any filter on colinearity. This accords with observation. Operons containing at least one gene gained by horizontal gene transfer (69% colinearity) are not significantly more ordered than all other operons (53% colinearity), *p* = 0.13. Conversely, operons with essential genes are unlikely to be gained by horizontal transfer according to the selfish operon hypothesis. As then expected, operons containing at least one essential gene (61.8% colinearity) are not less ordered than the rest of operons (60% colinearity), *p* = 0.9 (see Methods).

The finding that operonic gene order could be an adaptation against noise in protein levels leaves at least one paradoxical problem: although we see evidence for selection on gene order in operons, gene order within operons shows especially high evolutionary conservation, indicating strong purifying selection on local gene organization [34]. Indeed, a comparison of *E. coli* metabolic operons with information on operon structure and orthology in *Bacillus subtilis* (see Methods) revealed that 70% of *E. coli* operons could not be matched to conserved *B. subtilis* operons, either due to the absence of orthologs, or due to the fact that the orthologs are no longer located in the same transcription unit. In another 22% of the operons, the relative order of the orthologs was completely conserved, and we detected only five operons (8%) in which the relative position of orthologs had been rearranged. This freezing of gene order within operons may reflect the fact that intraoperonic inversions will place genes on the wrong strand. How can the two apparently contradictory findings be resolved? We speculate that the selection is not on order within the operon per se, but rather selection on successful establishment of operons. Imagine that A and B reside next to each other and a mutation occurs that permits them to be coded in a polycistronic transcript, i.e., operonization. If the operon is lowly expressed, our results suggest that selection for operonization will be stronger if the order is AB than if it is BA. Hence, a selective filter on operonization can explain the findings and be consistent with frozen operons after their establishment.

## Methods

### Mathematical Modelling

We considered a four-enzyme irreversible linear metabolic pathway coupled with operonic gene expression. The enzymes were assumed to operate according to standard Michaelis-Menten equations. All enzymes had the same velocities, turnover numbers, and *K_m_* values based on experimentally measured values of aspartate kinase I (Table S1). Cell generation time was 60 min, and all metabolites and enzymes were diluted accordingly (*D*). Initially, all metabolites had zero concentrations except for the substrate of the first enzyme, which was fixed at 1 mM. Operonic gene expression was modelled following the read-through operon model by Swain [17]; see Figure 2. Copasi version 4.4.28 was used to perform all simulations [35]. Stochastic simulations were carried out using a hybrid deterministic–stochastic simulation algorithm built into Copasi (“Hybrid Runge-Kutta”) to simulate gene expression and enzymatic reactions within one model (default parameter values were used with the exception of Runge-Kutta step size, which was set to 0.1).

### Dataset Compilation

Operonic gene order and metabolic pathways were extracted from EcoCyc [15] v10.5. We compiled a list of metabolic gene sets, in which each set consisted of genes belonging to the same pathway and encoded in the same operon. To enable the quantification of the extent of colinearity, we generated a list of nonredundant intraoperonic gene pairs with unambiguous metabolic pathway order (cyclic pathways were excluded), resulting in 321 gene pairs from 70 operons and 73 pathways (see Protocol S2). Data on *B. subtilis* operon structures were obtained from BioCyc [36] and DBTBS [37] (where information on a large number of experimentally characterized *B. subtilis* operons is available). Cross-species comparison of orthologs and chromosomal positions was performed using EcoCyc [15].

### Measuring Colinearity

Colinearity of a set of operons was measured as the ratio of the number of colinear pairs to the total number of gene pairs across all investigated operons. A gene pair was considered colinear if the gene located closer to the 5′ end of the operon encodes an enzyme operating earlier in the same pathway compared to the downstream gene.

To assess the statistical significance of colinearity in our dataset, we compared the observed level of colinearity to a distribution of colinearity values generated by randomizing gene order within each operon 100,000 times (*p*-values were calculated by *p* = (*R*+1)/(*N*+1), where *R* is the number of cases when the randomized level of colinearity is equal or greater than the observed level, and *N* is the number of randomizations).

### mRNA Abundance Profiles

Affymetrix microarray gene expression data were obtained from ref [38]. We used log_2_-transformed normalized expression profiles of wild-type K-12 MG1655 strain grown on M9 glucose medium under aerobic and anaerobic conditions [38]. For each gene, we calculated the average expression value based on three (aerobic) and four (anaerobic) data points. Transcript abundance level of operons was defined as the mean of expression values of the constituent genes.

To examine whether operons display a decreasing level of mRNA abundances from 5′ to 3′ end, we compiled a nonoverlapping set of gene strings from the whole *E. coli* genome, in which genes associated with a given string are always transcribed together. This resulted in 386 gene strings (2,199 within-string pairs) containing at least two genes with expression data. We counted the number of cases where the 5′ member of a gene pair has a higher expression level than the downstream gene (aerobic dataset: 1,274 pairs, anaerobic: 1,293 pairs) and compared those cases to the values obtained by randomizing the positions of within-string genes (*p*<10^−6^ for both conditions). A similar analysis was performed on our filtered set of metabolic operons (270 gene pairs in 65 operons; *p* = 0.0295 and *p* = 0.0224 for aerobic and anaerobic conditions, respectively).

We also determined for each operon whether its mRNA abundance profile shows a significant monotonic decrease from 5′ to 3′ end. The presence of a monotonically changing abundance was tested by linear trend analysis [28] (using gmodels R package). The direction of change was inferred from the Spearman rank correlation. After Bonferroni correction, we found 26 and 23 operons showing a significantly decreasing mRNA expression profile under aerobic and anaerobic conditions, respectively.

To quantify gene expression variability across environmental conditions, we used compiled expression data for 213 conditions [39]. We calculated the SD of the log_2_-transformed expression values for each gene, which is invariant under a multiplicative change [40]. Expression variability of operons was defined as the mean of SD values of the constituent genes. To examine whether expression variability of operons correlates with the degree of colinearity when controlling for mRNA abundance levels (as measured under aerobic and anaerobic glucose conditions), we used residuals from the linear regression of SD on expression level to classify operons into groups with higher or lower than average gene expression variability. Randomisation was employed to test whether the colinearity of these two groups were different. A similar procedure was followed to examine whether mRNA abundance of operons is associated with colinearity when expression variability is controlled for.

### Data on Horizontally Transferred Genes and Gene Essentiality

Genes that have undergone horizontal transfer into the *E. coli* lineage since its split from the *Vibrio* lineage were previously identified using parsimony analysis of gene presence and absence data [41]. Data on gene essentiality were obtained from a recent functional genomic study in *E. coli* K12, in which a systematic collection of in-frame, single-gene deletion mutants was constructed [42].

## Supporting Information

Figure S1 The impact on pathway productivity of swapping the position of two intraoperonic genes depends on their physical distance. The metabolic performance of every possible gene order of a four-gene operon was calculated by simulating the model deterministically, and three groups were defined based on the physical distance of the swapped genes (only those gene orders were compared in a pair-wise manner, which can be rearranged by swapping the position of one gene pair). Metabolic performance was defined here as the amount of end product accumulated during one cell-generation time after operon induction. Mean values and 95% confidence intervals are shown on the plot. We employed a randomization protocol to test whether the differences between mean values for groups 2 and 1, and for groups 3 and 2 are significant (*p* = 0.0001 based on 100,000 permutations of individual productivity differences between the groups).
(0.07 MB TIF)

Protocol S1 Compiling data on enzyme–enzyme regulatory interactions mediated by small molecules.
(0.03 MB DOC)

Protocol S2 Generation of a list of nonredundant intraoperonic gene pairs with unambiguous metabolic pathway order.
(0.03 MB DOC)

Table S1 Parameters and constants used in the mathematical models of metabolism and gene expression.
(0.05 MB DOC)

Table S2 Steady-state pathway flux with different operonic gene orders.
(0.03 MB DOC)

Table S3 Steady-state pathway flux with different operonic gene orders in the presence of polarity effects.
(0.03 MB DOC)

Table S4 Robustness of the deterministic simulation results to variations in substrate concentration and *K_m_*.
(0.04 MB DOC)

Table S5 Robustness of the stochastic simulation results to variations in substrate concentration.
(0.03 MB DOC)

## Abbreviations

SD: standard deviation

## References

[1] Hurst LD, Pal C, Lercher MJ (2004). The evolutionary dynamics of eukaryotic gene order.. Nat Rev Genet.

[2] Rison SC, Teichmann SA, Thornton JM (2002). Homology, pathway distance and chromosomal localization of the small molecule metabolism enzymes in Escherichia coli.. J Mol Biol.

[3] Salgado H, Moreno-Hagelsieb G, Smith TF, Collado-Vides J (2000). Operons in Escherichia coli: genomic analyses and predictions.. Proc Natl Acad Sci U S A.

[4] Pál C, Hurst LD (2004). Evidence against the selfish operon theory.. Trends Genet.

[5] Williams EJ, Bowles DJ (2004). Coexpression of neighboring genes in the genome of Arabidopsis thaliana.. Genome Res.

[6] Raj A, Peskin CS, Tranchina D, Vargas DY, Tyagi S (2006). Stochastic mRNA synthesis in mammalian cells.. PLoS Biol.

[7] Batada NN, Urrutia AO, Hurst LD (2007). Chromatin remodelling is a major source of coexpression of linked genes in yeast.. Trends Genet.

[8] Ebisuya M, Yamamoto T, Nakajima M, Nishida E (2008). Ripples from neighbouring transcription.. Nat Cell Biol.

[9] Rocha EP (2008). The organization of the bacterial genome.. Annu Rev Genet.

[10] Price MN, Huang KH, Arkin AP, Alm EJ (2005). Operon formation is driven by co-regulation and not by horizontal gene transfer.. Genome Res.

[11] Lawrence JG, Roth JR (1996). Selfish operons: horizontal transfer may drive the evolution of gene clusters.. Genetics.

[12] Martin FJ, McInerney JO (2009). Recurring cluster and operon assembly for Phenylacetate degradation genes.. BMC Evol Biol.

[13] Tamames J, Gonzalez-Moreno M, Mingorance J, Valencia A, Vicente M (2001). Bringing gene order into bacterial shape.. Trends Genet.

[14] Omelchenko MV, Makarova KS, Wolf YI, Rogozin IB, Koonin EV (2003). Evolution of mosaic operons by horizontal gene transfer and gene displacement in situ.. Genome Biol.

[15] Keseler IM, Collado-Vides J, Gama-Castro S, Ingraham J, Paley S (2005). EcoCyc: a comprehensive database resource for Escherichia coli.. Nucleic Acids Res.

[16] Zaslaver A, Mayo AE, Rosenberg R, Bashkin P, Sberro H (2004). Just-in-time transcription program in metabolic pathways.. Nat Genet.

[17] Swain PS (2004). Efficient attenuation of stochasticity in gene expression through post-transcriptional control.. J Mol Biol.

[18] Alpers DH, Tomkins GM (1965). The order of induction and deinduction of the enzymes of the lactose operon in E. Coli.. Proc Natl Acad Sci U S A.

[19] Alpers DH, Tomkins GM (1966). Sequential transcription of the genes of the lactose operon and its regulation by protein synthesis.. J Biol Chem.

[20] Nishizaki T, Tsuge K, Itaya M, Doi N, Yanagawa H (2007). Metabolic engineering of carotenoid biosynthesis in Escherichia coli by ordered gene assembly in Bacillus subtilis.. Appl Environ Microbiol.

[21] Ullmann A, Joseph E, Danchin A (1979). Cyclic AMP as a modulator of polarity in polycistronic transcriptional units.. Proc Natl Acad Sci U S A.

[22] Heinrich R, Klipp E (1996). Control analysis of unbranched enzymatic chains in states of maximal activity.. J Theor Biol.

[23] Klipp E, Heinrich R, Holzhutter HG (2002). Prediction of temporal gene expression. Metabolic opimization by re-distribution of enzyme activities.. Eur J Biochem.

[24] Elowitz MB, Levine AJ, Siggia ED, Swain PS (2002). Stochastic gene expression in a single cell.. Science.

[25] Cai L, Friedman N, Xie XS (2006). Stochastic protein expression in individual cells at the single molecule level.. Nature.

[26] Pál C, Papp B, Hurst LD (2001). Highly expressed genes in yeast evolve slowly.. Genetics.

[27] Drummond DA, Bloom JD, Adami C, Wilke CO, Arnold FH (2005). Why highly expressed proteins evolve slowly.. Proc Natl Acad Sci U S A.

[28] Quinn GP, Keough MJ (2002). Experimental design and data analysis for biologists..

[29] Newman JR, Ghaemmaghami S, Ihmels J, Breslow DK, Noble M (2006). Single-cell proteomic analysis of S. cerevisiae reveals the architecture of biological noise.. Nature.

[30] Fani R, Brilli M, Lio P (2005). The origin and evolution of operons: the piecewise building of the proteobacterial histidine operon.. J Mol Evol.

[31] Gutteridge A, Kanehisa M, Goto S (2007). Regulation of metabolic networks by small molecule metabolites.. BMC Bioinformatics.

[32] Schomburg I, Chang A, Ebeling C, Gremse M, Heldt C (2004). BRENDA, the enzyme database: updates and major new developments.. Nucleic Acids Res.

[33] Batada NN, Hurst LD (2007). Evolution of chromosome organization driven by selection for reduced gene expression noise.. Nat Genet.

[34] Rocha EP (2006). Inference and analysis of the relative stability of bacterial chromosomes.. Mol Biol Evol.

[35] Hoops S, Sahle S, Gauges R, Lee C, Pahle J (2006). COPASI–a COmplex PAthway SImulator.. Bioinformatics.

[36] Caspi R, Foerster H, Fulcher CA, Kaipa P, Krummenacker M (2008). The MetaCyc Database of metabolic pathways and enzymes and the BioCyc collection of Pathway/Genome Databases.. Nucleic Acids Res.

[37] Sierro N, Makita Y, de Hoon M, Nakai K (2008). DBTBS: a database of transcriptional regulation in Bacillus subtilis containing upstream intergenic conservation information.. Nucleic Acids Res.

[38] Covert MW, Knight EM, Reed JL, Herrgard MJ, Palsson BO (2004). Integrating high-throughput and computational data elucidates bacterial networks.. Nature.

[39] Price MN, Arkin AP, Alm EJ (2006). The life-cycle of operons.. PLoS Genet.

[40] Lewontin RC (1966). On the measurement of relative variability.. Syst Zool.

[41] Pál C, Papp B, Lercher MJ (2005). Adaptive evolution of bacterial metabolic networks by horizontal gene transfer.. Nat Genet.

[42] Baba T, Ara T, Hasegawa M, Takai Y, Okumura Y (2006). Construction of Escherichia coli K-12 in-frame, single-gene knockout mutants: the Keio collection.. Mol Syst Biol.

[43] Wilcox RR (2005). Introduction to robust estimation and hypothesis testing. 2nd edition.

