# Supplementary figures and images for: Stochasticity in Protein Levels Drives Colinearity of Gene Order in Metabolic Operons of Escherichia coli
Source: PLoS Biol. 2009 May 26;7(5):e1000115. doi: 10.1371/journal.pbio.1000115 (PMC2684527; doi:10.1371/journal.pbio.1000115)

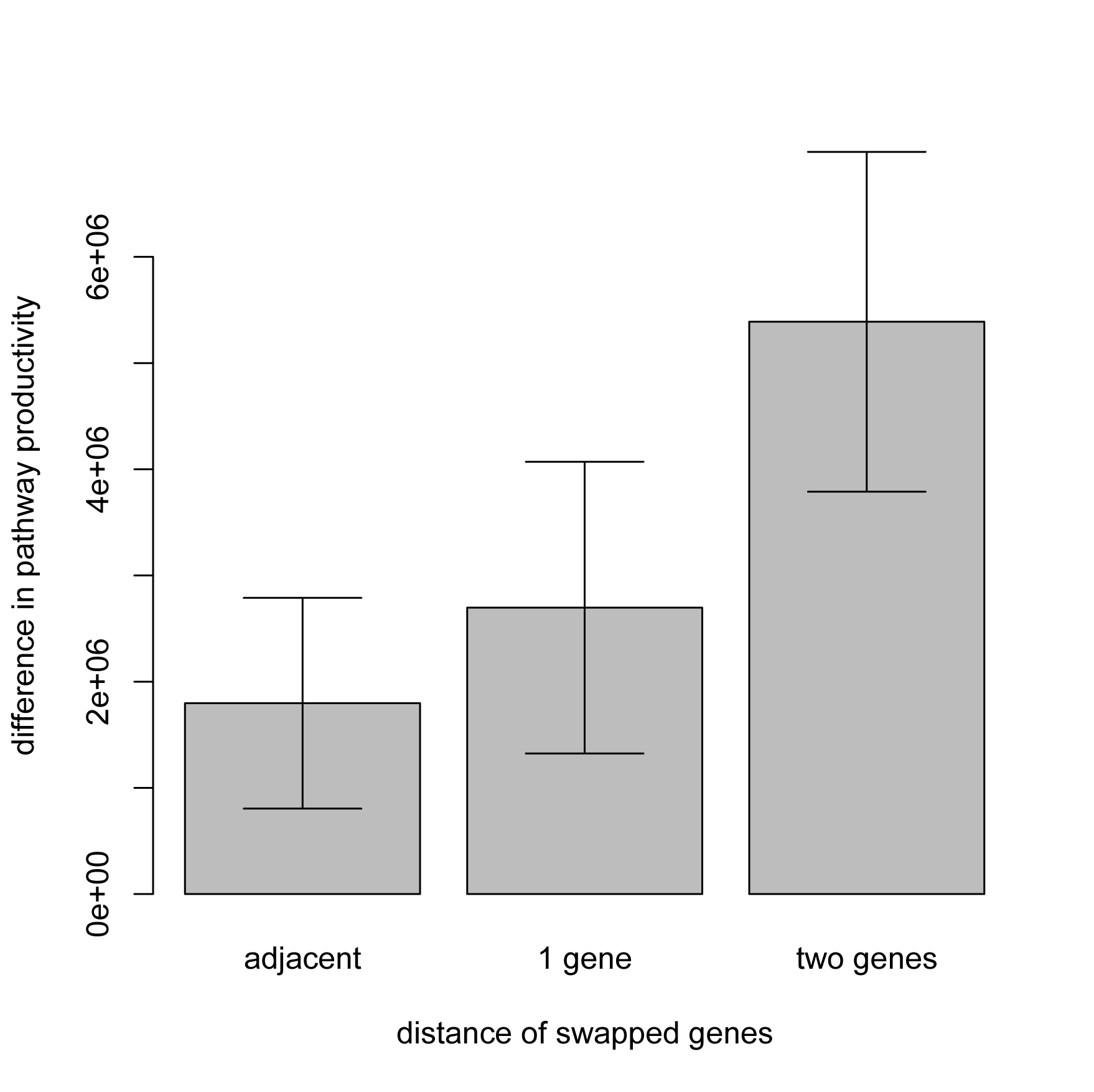

Supplement: Figure S1 — The impact on pathway productivity of swapping the position of two intraoperonic genes depends on their physical distance. The metabolic performance of every possible gene order of a four-gene operon was calculated by simulating the model deterministically, and three groups were defined based on the physical distance of the swapped genes (only those gene orders were compared in a pair-wise manner, which can be rearranged by swapping the position of one gene pair). Metabolic performance was defined here as the amount of end product accumulated during one cell-generation time after operon induction. Mean values and 95% confidence intervals are shown on the plot. We employed a randomization protocol to test whether the differences between mean values for groups 2 and 1, and for groups 3 and 2 are significant (p = 0.0001 based on 100,000 permutations of individual productivity differences between the groups). (0.07 MB TIF) [file pbio.1000115.s001.tif]
