# Supplementary material for: Stochasticity in Protein Levels Drives Colinearity of Gene Order in Metabolic Operons of Escherichia coli
Source: PLoS Biol. 2009 May 26;7(5):e1000115. doi: 10.1371/journal.pbio.1000115 (PMC2684527; doi:10.1371/journal.pbio.1000115)
Supplement: Protocol S1 — Compiling data on enzyme–enzyme regulatory interactions mediated by small molecules. (0.03 MB DOC) [file pbio.1000115.s002.doc]

**Supporting Protocol 1. Compiling data on enzyme – enzyme regulatory interactions mediated by small molecules.**

Using the EcoCyc database[1], and a dataset[2] based on the BRENDA database[3] we compiled a list of metabolite-level within-pathway regulatory interactions among enzymatic gene pairs encoded in the same operon (we considered cases where the product of one enzyme activates or inhibits another enzyme in the same pathway, except when the product is also the substrate of the regulated enzyme). We identified such regulatory interactions for 19 gene pairs encoded in 11 operons. To investigate whether the average gene distance between interacting gene pairs was different from chance expectations, we performed a randomization test in a way that in each round of randomization we only swapped the operonic positions of genes acting in the pathway investigated (i.e. the positions of genes with unrelated functions were preserved). The observed average gene distance was not significantly different from that obtained from randomizations (1.84 versus 2.07, P=0.234; the distance between adjacent genes was defined as 1). To examine whether the presence of intra-operonic regulatory interactions has an effect on the extent of colinearity, we compared the degree of colinearity in operons with known regulatory interactions (11 operons) to the rest of the dataset (59 operons). The statistical significance of this difference was assessed by comparing it to a distribution of differences obtained by randomly generating two samples with the same number of operons as in the original sets. Since operons with known regulatory interactions has significantly higher mRNA levels than the rest of operons and expression level correlates with the degree of colinearity, we repeated the above comparison after controlling for mRNA abundance. To control for mRNA levels, we selected the largest subset of the 59 operons without regulatory interactions in such a way that mRNA abundance in this group was no longer significantly different from the group of operons with known regulatory interactions (P=0.0504 and P=0.0516 for anaerobic and aerobic expression datasets, respectively). This resulted in subsets of 35 and 36 operons without regulatory interaction under anaerobic and aerobic conditions, respectively. Finally, we compared the degree of colinearity between these groups and the group of operon with reported regulatory interactions.

**References**

1. Keseler IM, Collado-Vides J, Gama-Castro S, Ingraham J, Paley S, et al. (2005) EcoCyc: a comprehensive database resource for Escherichia coli. Nucleic Acids Res 33: D334-337.

2. Gutteridge A, Kanehisa M, Goto S (2007) Regulation of metabolic networks by small molecule metabolites. BMC Bioinformatics 8: 88.

3. Schomburg I, Chang A, Ebeling C, Gremse M, Heldt C, et al. (2004) BRENDA, the enzyme database: updates and major new developments. Nucleic Acids Res 32 Database issue: D431-433.
