# Supplementary material for: Stochasticity in Protein Levels Drives Colinearity of Gene Order in Metabolic Operons of Escherichia coli
Source: PLoS Biol. 2009 May 26;7(5):e1000115. doi: 10.1371/journal.pbio.1000115 (PMC2684527; doi:10.1371/journal.pbio.1000115)
Supplement: Protocol S2 — Generation of a list of nonredundant intraoperonic gene pairs with unambiguous metabolic pathway order. (0.03 MB DOC) [file pbio.1000115.s003.doc]

**Supporting Protocol 2. Generation of a list of non-redundant intra-operonic gene pairs with unambiguous metabolic pathway order.**

We extracted information on operonic gene order and metabolic pathways from the EcoCyc database[1] version 10.5 (http://www.ecocyc.com). First, we compiled a list of metabolic gene sets, where each set comprised of genes belonging to the same pathway and encoded in the same operon (only sets with at least two genes acting at different reaction steps were further considered). To enable the quantification of the extent of colinearity, we generated a list of non-redundant intra-operonic gene pairs with unambiguous metabolic pathway order (e.g. cyclic pathways were excluded). This procedure involved the following steps: i) In case of hierarchically overlapping operons, we considered the operon containing the greatest number of examined genes. ii) Similarly, metabolic pathways can be part of superpathways, when the longer pathway contains all elements of the shorter one. In this situation, we considered the shortest pathway containing all the examined genes. In the case of non-hierarchically overlapping pathways, gene pairs were included only if their metabolic order was unambiguous. iii) In the case of branched pathways, we treated the different linear reaction series as different pathways (gene pairs belonging to overlapping parts of the pathways were counted only once). For simplicity, we refer to these unbranched linear reaction series as ”pathways”. iv) In some cases, an enzyme catalyses multiple reactions within the same pathway. If there is no examined gene between the two reaction steps, the metabolic order of any gene pair can be determined unambiguously. Otherwise, we considered all non-redundant intra-operonic gene pairs with unambigous pathway order. v) If more than one enzymatic gene is associated with the same reaction step (i.e. isoenzymes or enzyme complex subunits) we formed pairs for each associated gene separately. vi) In most cases, pathways in the Ecocyc database do not include the transporter importing the pathway’s susbtrate molecule. Therefore these transporter genes were manually added to our gene sets if encoded in the same operon. Overall, this procedure resulted in a list of 321 gene pairs from 70 operons and 73 pathways.

**References**

1. Keseler IM, Collado-Vides J, Gama-Castro S, Ingraham J, Paley S, et al. (2005) EcoCyc: a comprehensive database resource for Escherichia coli. Nucleic Acids Res 33: D334-337.
