# Supplementary material for: Stochasticity in Protein Levels Drives Colinearity of Gene Order in Metabolic Operons of Escherichia coli
Source: PLoS Biol. 2009 May 26;7(5):e1000115. doi: 10.1371/journal.pbio.1000115 (PMC2684527; doi:10.1371/journal.pbio.1000115)
Supplement: Table S1 — Parameters and constants used in the mathematical models of metabolism and gene expression. (0.05 MB DOC) [file pbio.1000115.s004.doc]

**Supporting Table 1. Parameters and constants used in the mathematical models of metabolism and gene expression**

| **Process / Constant** | **Parameters** |
| --- | --- |
| Enzyme turnover number | *k_cat_* = 50 s^-1^, approximated value based on empirical value measured for *E. coli* aspartate kinase I (ref [1]) |
| *K_m_* of enzymes | *K_m_* = 1 mM, approximated value based on empirical value measured for *E. coli* aspartate kinase I (ref [1]) |
| Dilution rate | *D* = 0.00019254 s^-1^ (based on 60 minutes cell cycle time) |
| Protein degradation rate | *k_degr_* = 6.42 * 10^-5^ s^-1^ (from ref [2]) |
| Protein dilution and degradation rate | α = 0.00025674 s^-1^ (α *= D + k_degr_*) |
| *E. coli* cell aqueous volume | *V* = 7 * 10^-16^ l (source : redpoll.pharmacy.ualberta.ca/CCDB/cgi-bin/STAT_NEW.cgi) |
| RNA polymerase binding to DNA (*f_0_*) and dissociation (*b_0_*) | *f_0_* = 0.42 s^-1^ (from ref [2]), see Fig 2., *b_0_* = 0.1 for highly expressed operons and *b_0_* = 1000 for lowly expressed operons |
| Transcription initiation rate | *k_0_* = 0.1 s^-1^ (from ref [2]), see Fig 2. |
| Formation (*v_0_*) and degradation (*mf_0_*) of ribosome binding site on mRNA | *v_0_* = 0.03 s^-1^, *mf_0_* = 0.114 s^-1^ (from ref [2]), see Fig 2. |
| Ribosome binding (*mf_1_*) and dissociation (*mb_1_*) | *mf _1_*= 4 s^-1^, *mb_1_* = 0.4 s^-1^ (from ref [2]), see Fig 2. |
| Translation rates | *k_1_* = 0.3 s^-1^ (from ref [2]), *v_1_* = 0.017 s^-1^ (fine tuned to achieve approx. 60 s delays between the appearance of consecutive gene products), see Fig 2. |
|  |  |

**References**

1. Chassagnole C, Fell DA, Rais B, Kudla B, Mazat JP (2001) Control of the threonine-synthesis pathway in Escherichia coli: a theoretical and experimental approach. Biochem J 356: 433-444.

2. Swain PS (2004) Efficient attenuation of stochasticity in gene expression through post-transcriptional control. J Mol Biol 344: 965-976.
