# Supplementary material for: Stochasticity in Protein Levels Drives Colinearity of Gene Order in Metabolic Operons of Escherichia coli
Source: PLoS Biol. 2009 May 26;7(5):e1000115. doi: 10.1371/journal.pbio.1000115 (PMC2684527; doi:10.1371/journal.pbio.1000115)
Supplement: Table S2 — Steady-state pathway flux with different operonic gene orders. (0.03 MB DOC) [file pbio.1000115.s005.doc]

**Supporting Table 2. Steady-state pathway flux with different operonic gene orders.**

We compared the results of two deterministic simulations of the metabolic model: one with perfectly colinear operonic gene order (ABCD) and another with anti-colinear gene order (DCBA). Fluxes through the last enzyme (*E*_4_) were compared under these two gene order configurations at a time point after which the concentrations of all pathway intermediates remained constant (at 8-digit accuracy).

| **Gene order** | **Flux through *E*_4_ (mmol * s^-1^)** |
| --- | --- |
| ABCD | 1.64033624*10^-16^ |
| DCBA | 1.64033624*10^-16^ |
|  |  |
