# Supplementary material for: Stochasticity in Protein Levels Drives Colinearity of Gene Order in Metabolic Operons of Escherichia coli
Source: PLoS Biol. 2009 May 26;7(5):e1000115. doi: 10.1371/journal.pbio.1000115 (PMC2684527; doi:10.1371/journal.pbio.1000115)
Supplement: Table S3 — Steady-state pathway flux with different operonic gene orders in the presence of polarity effects. (0.03 MB DOC) [file pbio.1000115.s006.doc]

**Supporting Table 3. Steady-state pathway flux with different operonic gene orders in the presence of polarity effects.**

We compared the results of two deterministic simulations of the metabolic model in the presence of polarity effects: one with perfectly colinear operonic gene order (ABCD) and another with anti-colinear gene order (DCBA). Polarity was included in the model by introducing degradation terms for ribosome-bound mRNA intermediates (*mT_2_, mT_3_, mT_4_*). This degradation parameter was set such that it resulted in a one third drop of enzyme levels for consecutive genes (i.e. it was set to one half of the translation rate). Because these degradation terms decrease total enzyme level, we adjusted model parameters such that the total enzyme level remained unchanged. Fluxes through the last enzyme (*E*_4_) were compared under these two gene order configurations at a time point after which the concentrations of all pathway intermediates remained constant (at 8-digit accuracy). The above procedure was repeated at both high and low expression levels (deterministic simulations in all cases). The simulations show that colinearity has a clear advantage in the presence of polarity effects and this advantage is greater when expression level is high (50% compared to 30.5%).

| **Gene order** | **Flux through *E*_4_ (mmol * s^-1^)** | |
| --- | --- | --- |
|  | **low expression** | **high expression** |
| ABCD | 1.5087192*10^-20^ | 1.61728822*10^-16^ |
| DCBA | 1.0479932*10^-20^ | 0.80871126*10^-16^ |
|  |  |  |
