# Supplementary material for: Stochasticity in Protein Levels Drives Colinearity of Gene Order in Metabolic Operons of Escherichia coli
Source: PLoS Biol. 2009 May 26;7(5):e1000115. doi: 10.1371/journal.pbio.1000115 (PMC2684527; doi:10.1371/journal.pbio.1000115)
Supplement: Table S4 — Robustness of the deterministic simulation results to variations in substrate concentration and Km. (0.04 MB DOC) [file pbio.1000115.s007.doc]

**Supporting Table 4. Robustness of the deterministic simulation results to variations in substrate concentration and Km.**

We repeated the deterministic simulations of the metabolic model with different substrate concentrations (*S_0_*) and Michaelis-Menten constants.

| **Substrate** | **Michaelis-Menten** | **Relative advantage of colinearity** | |
| --- | --- | --- | --- |
| **concentration** | **constant** | **after 1 generation** | **after 50 generations** |
| 0.01 mM | 1 | 8.64% | 0.0995% |
| 0.1 mM | 1 | 8.63% | 0.0996% |
| 1 mM | 1 | 8.49% | 0.0990% |
| 10 mM | 1 | 5.12% | 0.0812% |
| 100 mM | 1 | 2.45% | 0.0490% |
| 1 mM | 0.01 | 1.11% | 0.0517% |
| 1 mM | 0.1 | 7.48% | 0.0926% |
| 1 mM | 10 | 8.64% | 0.0970% |
| 1 mM | 100 | 10.38% | 0.0878% |
|  |  |  |  |
