# Supplementary material for: Stochasticity in Protein Levels Drives Colinearity of Gene Order in Metabolic Operons of Escherichia coli
Source: PLoS Biol. 2009 May 26;7(5):e1000115. doi: 10.1371/journal.pbio.1000115 (PMC2684527; doi:10.1371/journal.pbio.1000115)
Supplement: Table S5 — Robustness of the stochastic simulation results to variations in substrate concentration. (0.03 MB DOC) [file pbio.1000115.s008.doc]

**Supporting Table 5. Robustness of the stochastic simulation results to variations in substrate concentration.**

We repeated the stochastic simulations of the metabolic model with different substrate concentrations (*S_0_*) and found that the relative advantage of colinearity after 50 cell generations is consistently ~ 30 – 100-fold higher at low expression level than at high expression.

| **Substrate concentration** | **Relative advantage of colinearity** | |
| --- | --- | --- |
|  | **low expression** | **high expression** |
| 0.01 mM | 3.48% | 0.1% |
| 1 mM | 4.65% | 0.1% |
| 100 mM | 5.45% | 0.05% |
|  |  |  |
